# Potential Therapeutic Applications of N-Cadherin Antagonists and Agonists

**DOI:** 10.3389/fcell.2022.866200

**Published:** 2022-03-03

**Authors:** Orest W. Blaschuk

**Affiliations:** Zonula Incorporated, Kirkland, QC, Canada

**Keywords:** N-cadherin, adherens junctions, ADH-1, biomaterials, fibrosis, cancer, tissue regeneration, vasculature

## Abstract

This review focuses on the cell adhesion molecule (CAM), known as neural (N)-cadherin (CDH2). The molecular basis of N-cadherin-mediated intercellular adhesion is discussed, as well as the intracellular signaling pathways regulated by this CAM. N-cadherin antagonists and agonists are then described, and several potential therapeutic applications of these intercellular adhesion modulators are considered. The usefulness of N-cadherin antagonists in treating fibrotic diseases and cancer, as well as manipulating vascular function are emphasized. Biomaterials incorporating N-cadherin modulators for tissue regeneration are also presented. N-cadherin antagonists and agonists have potential for broad utility in the treatment of numerous maladies.

## Introduction

Cell adhesion molecules (CAMs) regulate cell adhesion, division, survival, differentiation, shape, and migration. Tissues are molded by the homotypic and heterotypic intercellular interactions of CAMs. Numerous intracellular signaling pathways are triggered when single cells adhere to one another. These activated pathways cause alterations in gene expression and subsequently impact cell behavior. CAMs are directly involved in many diseases (*e.g.,* cancer, fibrosis). Modulators of CAM function can consequently be developed to serve as therapeutics. This review focuses on a particular CAM, known as neural (N)-cadherin (CDH2). Inhibitors (*i.e*., antagonists) and stimulators (*i.e.,* agonists) of N-cadherin function are discussed, as well as their potential to serve as therapeutics for various diseases and promoters of tissue regeneration. The complexity of CAM function is revealed through the study of N-cadherin antagonist and agonist biological activity.

### N-Cadherin

Cadherins are calcium-dependent cell adhesion molecules, meaning that they bind calcium and require the presence of this ion to function (Hulpiau and van Roy 2008; Blaschuk 2015; Gul et al., 2017). There are 114 different human cadherins which have been divided into 11 subfamilies (Hulpiau et al., 2013, Gul et al.*,* 2017). They have varying tissue distributions. For example, neural (N)-cadherin is usually expressed by mesenchymal, neural, endothelial, and poorly differentiated cancer cells, whereas epithelial (E)-cadherin (CDH1) is expressed by epithelial cells and well-differentiated carcinomas (Tsuchiya et al., 2006; Mrozik et al., 2018; Derynck and Weinberg 2019). It is important to note that cells can express multiple cadherins. For example, endothelial cells express N-cadherin and vascular endothelial (VE)-cadherin (CDH5) (Colás-Algora and Millán 2019), whereas mesenchymal cells display N-cadherin and osteoblast (OB)-cadherin (CDH11) (Black et al., 2018). N-cadherin antagonists can inhibit the adhesion of cells displaying multiple cadherins (Alexander et al., 1993; Erez et al., 2004; Jiang et al., 2020).

N-cadherin is a member of the Type I cadherin subfamily. It is a single pass transmembrane glycoprotein containing extracellular (EC), transmembrane and cytoplasmic (CP) domains (Harrison et al., 2011; Blaschuk 2015; Mrozik et al., 2018). Type I cadherin monomers exist in different states in the plasma membrane and at intercellular adhesive contacts, where they function as homophilic and heterophilic CAMs (Harrison et al., 2011; Bunse et al., 2013; Vendome et al., 2014; Blaschuk 2015; Mrozik et al., 2018). The monomers apparently interact within the plane of the plasma membrane (referred to as *cis* interactions) and with identical monomers on the surface of apposing cells (referred to as *trans* interactions). These *cis* and *trans* interactions involve the cell adhesion recognition (CAR) sequence Histidine–Alanine–Valine (His79–Ala80–Val81) which is found towards the terminus of all Type I cadherin EC1 domains. This CAR sequence was predicted to be directly involved in mediating Type I cadherin-dependent cell adhesion over 3 decades ago (Blaschuk et al., 1990a; Blaschuk et al., 1990b; Blaschuk 2015). Later studies suggested that the His79 and Val81 residues facilitate *cis* interactions, whereas the Ala80 residue is involved in *trans* interactions (Harrison et al., 2011; Bunse et al., 2013; Blaschuk 2015). The Ala80 residue is a constituent of a hydrophobic pocket into which docks a tryptophan (Trp2) found at the N-terminus of all Type I cadherin EC1 domains, thus promoting intercellular adhesion.

Only the Type I cadherins contain a hydrophobic pocket involving the CAR sequence, His-Ala-Val (HAV) and a single Trp residue at the N-terminus of their EC domains. Other cadherin family members engage using similar, but distinct mechanisms. For example, the Type II cadherins (*e.g.*, osteoblast (OB)-cadherin) harbor two Trp residues in the second and fourth positions of their EC domains (Trp2 and Trp4) which mediate *trans* interactions between monomers (Patel et al., 2006; Hulpiau et al., 2013; Brasch et al., 2011, 2018). Consequently, Type II cadherins contain a larger hydrophobic binding pocket than the Type I cadherins to facilitate these interactions. It is therefore possible to design antagonists specific for Type I cadherins that do not inhibit intercellular adhesion mediated by other types of cadherins (Devemy and Blaschuk 2008, 2009).

### N-Cadherin-Associated Intracellular Proteins and Their Signaling Pathways

N-cadherin-mediated cell adhesion activates many intracellular signaling pathways governing cell proliferation, apoptosis, migration, and differentiation (Kourtidis et al., 2013; Krishnamurthy and Kurzrock, 2018; Mrozik et al., 2018; Flinn et al., 2020; Young et al., 2021). The N-cadherin interactome is dependent on cell context (Li et al., 2019). For example, there are at least 350 proteins in the cardiomyocyte N-cadherin interactome. Most of these proteins are intracellular adaptor proteins. The CP domain of N-cadherin (and other Type I cadherins) binds directly to two intracellular proteins, β-catenin (Hulpiau et al., 2013; Shao et al., 2017; Adhikari et al., 2018) and p120 (Kourtidis et al., 2013; Hong et al., 2016, Li et al., 2019). These two proteins regulate the strength of cadherin-mediated intercellular adhesion, collective cell migration and gene expression (Nagafuchi and Takeichi 1988; McLachlan and Yap 2007; Nelson 2008; Mrozik et al., 2018; Young et al., 2021). They bind to different sites on the CP domain. A juxta-membrane site on the CP domain associates with p120, while a site at the carboxy-terminus of this domain binds β-catenin (Kourtidis et al., 2013; Adhikari et al., 2018; Mrozik et al., 2018).

The intracellular protein, β-catenin links Type I cadherins to the F-actin cytoskeleton via α-catenin (Hulpiau et al., 2013; Shao et al., 2017; Adhikari et al., 2018). Two actin-binding proteins that interact with α-catenin are vinculin (Bachir et al., 2017; Bays and DeMali 2017) and afadin (Li et al., 2019). All three of these actin-binding, adaptor proteins appear to be necessary for the formation of stable, cadherin-containing intercellular adhesive junctions (also see discussion below on intercellular junctions) (Valenta et al., 2012; Bays and DeMali 2017, Li et al., 2019). Vinculin is also involved in promoting cell-extracellular matrix (ECM) adhesive interactions mediated by integrins (Bachir et al., 2017; Bays and DeMali 2017). Afadin interacts directly with the cytoplasmic domain of calcium-independent, immunoglobulin-like cell adhesion molecules called nectins (Niessen 2007; Shimono et al., 2012; Huxham et al., 2021). Although nectins do not directly associate with Type I cadherins *in vivo*, they promote the formation of intercellular junctions (Niessen 2007).

In addition to regulating cell adhesion as a component of the plasma membrane Type I cadherin/β-catenin/α-catenin complex, β-catenin is involved in several intracellular signaling pathways, such as the canonical Wnt/β-catenin (Bachir et al., 2017; Gul et al., 2017; Krishnamurthy and Kurzrock, 2018) and Hippo/Yap (Flinn et al., 2020) pathways.

In the canonical Wnt/β-catenin pathway, β-catenin is targeted by a cytoplasmic destruction complex composed of the scaffold proteins, Axin and adenomatous polyposis *coli* (APC), as well as two serine/threonine kinases, casein kinase 1 (CK1) and glycogen synthase kinase 3β (GSK3β) (Jiang 2017; Santiago et al., 2017; Krishnamurthy and Kurzrock, 2018; Houschyar et al., 2019). CK1 and GSK3β phosphorylate β-catenin in the destruction complex. Phosphorylated β-catenin is then ubiquitinated and degraded.

The phosphorylation state of β-catenin is regulated by the Wnt family of secreted, cysteine-rich glycoproteins (composed of 19 members in mammals) that are known to play a pivotal role in development (Mikels and Nusse 2006; MacDonald et al., 2009; Hua et al., 2018; Krishnamurthy and Kurzrock, 2018) and cancer stem cell survival (Katoh 2017). The destruction complex is recruited to the plasma membrane upon Wnt binding to a receptor complex composed of Frizzled (Fz) and low density lipoprotein receptor-related protein 5/6 (LRP5/6) (Jiang 2017; Santiago et al., 2017; Krishnamurthy and Kurzrock, 2018; Houschyar et al., 2019). Mammals have 10 Fz receptors and two LRPs (MacDonald et al., 2009). The Wnt receptor and destruction complexes are linked together via the intracellular protein, Dishevelled (Dsh) (Hua et al., 2018; Sharma et al., 2018; Houschyar et al., 2019). Linkage of the two complexes prevents β-catenin phosphorylation. Consequently, β-catenin accumulates in the cytoplasm and subsequently translocates to the nucleus where it is capable of acting as a co-activator of gene transcription by forming complexes with transcription factors, such as members of the LEF (lymphoid enhancer factor-1)/Tcf (T cell factor) family (LEF-1, Tcf1, Tcf3 and Tcf4), Dsh, androgen receptor, ESE1 (epithelial specific ETS1) and tribbles pseudo-kinase 3 (TRIB3) (Cadigan and Waterman 2012; Santiago et al., 2017; Sharma et al., 2018; Mulholland et al., 2005; Yang and Lee 2016; Hua et al., 2019, respectively). These complexes regulate the transcription of a variety of genes (e.g., c-myc, cyclin D-1, Axin2). It is important to note that β-catenin does not bind directly to DNA (MacDonald et al., 2009; Aloysius et al., 2018). Instead, β-catenin displaces co-repressors of transcription (e.g., Tle) and forms complexes with DNA-binding transcription factors (e.g., Tcf3) at target gene promoters (Aloysius et al., 2018; Ramakrishnan et al., 2018).

Type I cadherins can also alter gene expression patterns in response to mechanical stimuli. The ability of N-cadherin to act as a mechanosensor is dependent on the canonical Wnt/β-catenin and Hippo/Yap pathways (Cosgrove et al., 2016; Flinn et al., 2020; Jiang et al., 2020; Pobbati and Hong 2020; Szulzewsky et al., 2021). The distribution of the oncoprotein, Yap within the cell can be membranous, cytoplasmic, or nuclear depending on whether it is phosphorylated. Yap (Yes-associated protein) is a transcriptional co-regulator that binds to β-catenin and subsequently regulates gene transcription via interaction with TEAD transcription factors. Tcf may also be a component of this complex. The Hippo kinase pathway controls Yap activity. This pathway is activated by mechanical stimuli (e.g., N-cadherin-mediated cell adhesion) resulting in phosphorylation of Yap, preventing it from relocating to the nucleus, thus abrogating transcriptional activity. Further studies are required to obtain a detailed understanding of this Type I cadherin/β-catenin/Yap pathway (Jiang et al., 2020).

In summary, cellular β-catenin exists in three pools: 1) plasma membrane; 2) cytoplasmic; and 3) nuclear. The cellular localization of β-catenin is dependent on its state of phosphorylation which is indirectly influenced by Wnt binding to a heterodimeric receptor complex composed of Fz and LRP5/6. The Hippo/Yap pathway may impact these pools.

Similar to β-catenin, the intracellular, Type I cadherin-binding protein, p120 also exists in three cellular pools (Keil et al., 2013; Kourtidis et al., 2013; Hong et al., 2016; Gul et al., 2017). It is a member of the armadillo (ARM) family of cadherin-binding proteins which includes β-catenin (Hulpiau et al., 2013; Keil et al., 2013; Kourtidis et al., 2013; Gul et al., 2017). Members of this family all possess variable numbers of tandem repeats (termed ARM repeats), each containing approximately 40 amino acids (Shapiro and Weis 2009; Hulpiau et al., 2013; Keil et al., 2013). The ARM repeats mediate protein-protein interactions (e.g., p120 binding to the Type I cadherin CP domain).

N-cadherin clustering and function are controlled by p120 (Taulet et al., 2009; Hong et al., 2016; Mrozik et al., 2018; Gritsenko et al., 2020). In addition, p120 regulates N-cadherin endocytosis and degradation. The molecular mechanisms regulating formation of N-cadherin-p120 heterodimers are not fully understood, but changes in the phosphorylation of p120 have been reported to modulate the interaction between this intracellular protein and Type I cadherins (Hong et al., 2016; Kota et al., 2019). Studies have shown that phosphorylation of selected tyrosine residues on p120 correlates with an increased binding affinity for Type I cadherins.

In addition to binding Type I cadherins, p120 also interacts with Rho GTPase family members, thereby regulating their activity and influencing actin cytoskeletal dynamics (Menke and Giehl 2012; Kourtidis et al., 2013; Kota et al., 2019; Gritsenko et al., 2020). Collective cell migration is therefore modulated by Type I cadherin-mediated intercellular adhesion (Gritsenko et al., 2020).

The serine/threonine kinase CK1ε phosphorylates p120 in response to stimulation by Wnt (see preceding discussion of the Wnt/β-catenin signaling pathway) (Jiang 2017; Pierre et al., 2019). Phosphorylated p120 translocates to the nucleus where it interacts with Kaiso, a repressor of gene transcription. Notably, Kaiso represses the transcription of genes encoding epithelial (E)-cadherin, β-catenin, Wnt11, matrilysin, miR-200 and cyclin D1. Paradoxically, this transcription factor is also capable of activating gene transcription. The switch from repressor to activator is currently thought to be dependent on the state of Kaiso SUMOylation. SUMOylated, Kaiso is believed to act as an activator, whereas deSUMOylated Kaiso functions as a repressor.

Kaiso is a positive regulator of c-myc, BRCA1, TGFβ1 and two receptor gene expression (Pierre et al., 2019). BRCA1 is involved in DNA repair of double-strand breaks (Macedo et al., 2019), whereas c-myc is a transcription factor regulating many biological processes, such as cell proliferation, genomic integrity, angiogenesis, metabolism and apoptosis (Elbadawy et al., 2019). TGFβ (the ligand for TGFβ1 and two receptors) is a key regulator of the biological process by which epithelial cells undergo morphogenetic changes, lose their apico-basal polarity, and enter a mesenchymal state where they are migratory and invasive (Nieto et al., 2016). This process is known as the epithelial-mesenchymal transition (EMT) and occurs during embryogenesis and oncogenesis. Notably, E- and N-cadherin are markers for epithelial and mesenchymal cell states, respectively (Derynck and Weinberg 2019). TGF-β signaling ultimately results in the activation of three families of transcription factors (ZEB, Snail and Twist) that down-regulate expression of epithelial genes such as E-cadherin and up-regulate expression of mesenchymal genes such as N-cadherin (Nieto et al., 2016; Goossens et al., 2017; Derynck and Weinberg 2019; Fardi et al., 2019). In summary, p120 influences the process of EMT by directly engaging with cadherins, regulating cytoskeletal dynamics and stimulating mesenchymal gene expression.

Intercellular adhesion mediated by N-cadherin triggers multiple intracellular signaling pathways resulting in profound morphogenetic changes in the adherent cells. The effects of N-cadherin antagonists and agonists on cells will predictably be complex and dependent on cell type.

### Intercellular Junctions Containing N-Cadherin

Intercellular junctions are protein-rich, lipid-poor plasma membrane domains formed between apposed adherent cells. They are complex, multi-protein structures that directly promote cell-cell adhesion and transduce stimuli from the external environment into the cell (Erez et al., 2005; Charras and Yap 2018; Li et al., 2019; Flinn et al., 2020; Young et al., 2021). Adherens junctions (AJs) are a diverse group of intercellular junctions (Erez et al., 2005; Franke et al., 2009, Li et al., 2019). They contain Type I cadherins (e.g., N-cadherin) and various other proteins (depending on the cell type) in their membranous domain, as well as β-catenin, α-catenin, p120 and other proteins (depending on the cell type) in a sub-membranous, electron dense, cytoplasmic plaque attached to microfilaments of the cytoskeleton. AJs can be formed by homotypic and heterotypic cell aggregates (Volk and Geiger 1986a; Volk et al., 1987; Miyatani et al., 1989; Mora et al., 2012). Heterotypic cell contacts contain AJs with either one, or two different Type I cadherins, whereas homotypic cell AJs contain one.

There are many morphologically distinct types of intercellular junctions containing N-cadherin (Franke et al., 2009; Domke et al., 2014; Vite and Radice 2014; Mizutani et al., 2021). The size and shape of Type I cadherin-containing intercellular junctions is likely regulated by p120 interacting with Rho GTPases, thereby influencing actin dynamics and cadherin clustering (Anastasiadis and Reynolds 2001; Ting et al., 2012; Kourtidis et al., 2013; Mrozik et al., 2018; Gritsenko et al., 2020).These junctions usually contain other CAMs in addition to N-cadherin (e.g., nectins, desmogleins) and associated intracellular proteins (e.g., afadins, catenins, protein tyrosine kinases and phosphatases), as well as growth factor receptors (e.g. FGF receptor) (Takai and Nakanishi 2003; Erez et al., 2005; McLachlan and Yap 2007; Niessen 2007; Franke et al., 2009; Shimono et al., 2012; Mrozik et al., 2018, Li et al., 2019). The diverse intercellular junctions enable adult tissues to maintain their integrity in the absence of N-cadherin activity. Indeed, antagonists of N-cadherin are not toxic in humans and other animals (Blaschuk 2015).

### Biological Properties of N-Cadherin Antagonists

Studies utilizing N-cadherin antagonists have been ongoing for over 3 decades (Blaschuk 2015). The responses of different cell types to these antagonists are diverse, as the N-cadherin interactome is large and subject to manipulation by a variety of stimuli (e.g., growth factors). The composition of CAMs displayed by a given cell type is also variant, thereby resulting in differential effects of N-cadherin antagonists on intercellular adhesion, and the intracellular signaling pathways associated with this process.

The first N-cadherin antagonists were monoclonal antibodies (Mabs) capable of inhibiting cell-cell adhesion (Volk and Geiger 1986b; Hatta and Takeichi 1986; Blaschuk 2015). Studies have subsequently shown that Mabs directed against the N-cadherin EC domain can inhibit cancer cell proliferation and invasiveness *in vitro* and *in vivo* (Tanaka et al., 2010; Mrozik et al., 2018). These and other observations have given rise to the hypothesis that N-cadherin antagonists may be useful anti-cancer drugs (see next section). Furthermore, Mabs directed against N-cadherin block the differentiation of cells, such as osteoblasts (Hay et al., 2000; Marie 2002) and mesenchymal stem cells (MSC) (Oberlender and Tuan 1994; Tuan 2003) underlining the ability of CAMs to modulate gene expression.

Short, synthetic linear peptides containing the Type I cadherin CAR sequence, His-Ala-Val (AHAVSE and LRAHAVDVNG) were then shown to be capable of disrupting N-cadherin-mediated neurite outgrowth on astrocytes (Blaschuk et al., 1990b; Chuah et al., 1991). Other papers followed demonstrating the ability of synthetic peptides harboring the CAR sequence to inhibit a variety of N-cadherin-mediated processes, such as myoblast fusion (Mege et al., 1992) and bone marrow osteoprogenitor stromal cell adhesion (Cheng et al., 1998).

Phage display technology was used to identify additional amino acid sequences capable of binding to N-cadherin (Devemy and Blaschuk 2008; Blaschuk and Devemy 2013). All the peptides (12-mers) that were identified harbored a Trp residue in the second position from the N-terminus (e.g., SWTLYTPSGQSK). Similarly, all type I cadherin ectodomains harbor a Trp residue in the second position from the N-terminus. As previously discussed, Type I cadherin-mediated intercellular adhesion involves the docking of a cadherin monomer Trp2 residue into a hydrophobic pocket (containing the HAV sequence) within a cadherin monomer ectodomain on the surface of an opposing cell. The peptide SWTLYTPSGQSK inhibited N-cadherin-dependent endothelial cell adhesion and tube formation *in vitro*.

A family of synthetic cyclic peptides containing the cadherin CAR sequence HAV was first described by Blaschuk and Gour (Blaschuk and Gour 2000). The most utilized N-cadherin antagonist of this family is the synthetic cyclic peptide CHAVC (also known as ADH-1 and Exherin) (Blaschuk 2015). This cyclic peptide has been shown to affect the N-cadherin-mediated adhesion, migration, proliferation, and apoptosis of a variety of cell types *in vitro*. For example, ADH-1 inhibits N-cadherin-dependent neurite outgrowth on fibroblasts (Williams et al., 2000), as well as Schwann cell (Wilby et al., 1999) and oligodendrocyte (Schnädelbach et al., 2000) adhesion to, and migration on astrocytes. ADH-1 has also been shown to inhibit bovine capillary endothelial cell (BCEC), murine capillary endothelial cell (MCEC) (Erez et al., 2004), granulosa cell (Makrigiannakis et al., 1999) and skin fibroblast adhesion (Jiang et al., 2020).

The loss of N-cadherin-mediated intercellular adhesion can lead to activation of apoptosis (Mrozik et al., 2018). For example, ADH-1 stimulates EC (Erez et al., 2004), granulosa (Makrigiannakis et al., 1999), neuroblastoma (Lammens et al., 2012) and vascular smooth muscle cell (VSMC) (Lyon et al., 2010) apoptosis *in vitro*. This effect of ADH-1 is thought to involve suppression of the phosphatidylinositide-3-kinase (PI3K)/Akt kinase/Bad pathway (Mrozik et al., 2018). The precise molecular mechanisms by which N-cadherin antagonists induce apoptosis remain to be clarified. In particular, the effects of these antagonists on the well-established association of the FGF receptor (the membrane anchor of the PI3K/Akt/Bad pathway) with N-cadherin in the plane of the plasma membrane (Nguyen and Mege 2016; Kon et al., 2019; Quintanal-Villalonga et al., 2020) needs to be thoroughly examined.

ADH-1 has been shown to inhibit cell migration. For example, fibroblasts present in the subcutaneous fascia of mouse skin form migratory, N-cadherin-dependent cell collectives (connected by AJs) in response to injury (i.e., deep skin cuts). ADH-1 prevents the formation of these cell collectives and their migratory behavior (Jiang et al., 2020). This N-cadherin antagonist is thought to influence formation and migration of cell collectives by indirectly regulating p120/Rho GTPase activity (Gritsenko et al., 2020).

ADH-1 inhibits tumor growth in pre-clinical animal models (Shintani et al., 2008; Mrozik et al., 2018). It has also been examined in phase I clinical trials for the treatment of solid cancers (Beasley et al., 2009; Perotti et al., 2009). ADH-1 was well-tolerated and showed potential as an antitumor drug. This is the only N-cadherin antagonist to enter clinical trials to date. The use of ADH-1 as an anti-cancer therapeutic is discussed in more detail below.

Numerous non-peptidyl peptidomimetics of ADH-1 have been discovered, but the biological properties of only one (LCRF-0006, 5-[(3,4-dichlorobenzyl)sulfanyl]-4H-1,2,4-triazol-3-amine) have been extensively studied (Gour et al., 2007; Mrozik et al., 2020). The N-cadherin antagonist LCRF-0006 inhibits neurite outgrowth and bone marrow endothelial cell (BMEC) adhesion *in vitro*. LCRF-0006 is also capable of disrupting BMEC monolayers, preventing endothelial tube formation in Matrigel, and disrupting mature endothelial tubes (Mrozik et al., 2020). LCRF-0006 did not cause BMEC apoptosis, in contrast to ADH-1. This discrepancy could be caused by the different types of ECs that were used to evaluate the apoptotic effects of the N-cadherin antagonists (BCEC and MCEC, as opposed to BMEC). This observation indicates that not all types of ECs respond similarly to N-cadherin antagonists.

LCRF-0006 is well-tolerated by mice. Treatment of mice with LCRF-0006 does not have obvious effects on bone blood vessel morphology, hematopoiesis, and N-cadherin expressing mesenchymal stromal cells, osteoblasts, and osteoprogenitors (Mrozik et al., 2020). Significantly, this N-cadherin antagonist did increase multiple myeloma (MM) tumor vascular permeability, suggesting that N-cadherin antagonists could facilitate drug delivery. The effects of N-cadherin antagonists on vasculature will be discussed further in a following section.

Non-peptidyl peptidomimetics of the Trp-containing, N-cadherin amino-terminus have been discovered and shown to inhibit N-cadherin-mediated intercellular adhesion (Vaisburg and Blaschuk 2019). One of these N-cadherin antagonists, designated compound 15, (S)-1-(3,4-Dichlorophenoxy)-3-(4-((S)-2-hydroxy-3-(4-methoxyphenoxy)propylamino)piperidin-1-yl)propan-2-ol causes Panc-1 human pancreatic cancer cell, U87MG human glioblastoma multiforme cell (GBM), fibroblast, cancer-associated fibroblast (CAF) and fibroblast-like synoviocyte (FLS) death (Blaschuk 2020; Smits et al., 2020). Normal human astrocytes are not affected by this compound, indicating that not all cell types are killed by this N-cadherin antagonist (Smits et al., 2020). Compound 15 was found to be well-tolerated by mice (Blaschuk 2020).

The preceding discussion indicates that N-cadherin antagonists have a wide variety of therapeutic applications. Some of these will be considered in the next section.

## Prospective Therapeutic Applications of N-Cadherin Antagonists

### N-Cadherin Antagonists for the Treatment of Fibroblast-Based Diseases

Fibroblasts are a heterogenous population of cells with different subtypes expressing variable levels of N-cadherin (Angelucci et al., 2012; Janson et al., 2013; Konstantinova and Pearce 2015; Jiang et al., 2020; Ascensión et al., 2021; Plikus et al., 2021). Inhibition of N-cadherin function has been shown to decrease fibroblast adhesion, migration, proliferation, survival, and differentiation (Konstantinova and Pearce 2015; Black et al., 2018; Blaschuk 2020; Jiang et al., 2020), as well as collagen synthesis and secretion by these cells (Konstantinova and Pearce 2015).

The excessive deposition of ECM proteins (e.g., collagen and fibronectin) by activated fibroblasts and myofibroblasts results in thickening and hardening of tissues (i.e., fibrosis) (Knuppel et al., 2017; Huang et al., 2021; Plikus et al., 2021). Tissue fibrosis is involved in the genesis of many diseases. For example, idiopathic pulmonary fibrosis (IPF) is believed to be initiated by damage to the lung alveolar epithelium from chronic inflammatory processes, infections, and environmental agents (Knuppel et al., 2017; Black et al., 2018; Chanda et al., 2019). Immune cells (e.g., macrophages) are recruited to the site of epithelial damage. They secrete TGF-β (a key, but not the only, mediator of fibrosis) which stimulates fibroblasts to differentiate into myofibroblasts. These cells proliferate and secrete collagen, which accumulates in the lungs as disorganized fibrils causing scar formation and loss of tissue elasticity. Inhibition of fibroblast function, differentiation and survival utilizing N-cadherin antagonists may thus be an effective anti-fibrotic strategy for treating pulmonary diseases such as IPF (Konstantinova and Pearce 2015; Black et al., 2018; Blaschuk 2020).

Fibroblasts are also intimately involved in the formation of scars resulting from the repair of skin wounds (Correa-Gallegos et al., 2019; Jiang et al., 2020; Jiang et al., 2021). Scar formation involves a subpopulation of fibroblasts (designated engrailed-past fibroblasts) residing in the subcutaneous fascia of the skin which are activated upon injury. These cells up-regulate N-cadherin, aggregate, and migrate as cell collectives into the wound where they deposit collagen giving rise to scars (Jiang et al., 2020). The N-cadherin antagonist ADH-1 inhibits these processes. Based on these observations, N-cadherin antagonists could be envisioned as therapeutics for the reduction of scar formation. The hypothesis has been proposed that fibroblast aggregation and migration is a general response to tissue injury. This hypothesis emphasizes the need to thoroughly investigate the biological effects of N-cadherin antagonists.

The microenvironment of tumors contains a heterogenous population of cells that are intimately associated, and reciprocally communicating with cancer cells, called cancer-associated fibroblasts (CAFs) (Öhlund et al., 2017; Nurmik et al., 2020; Sahai et al., 2020). These cells express N-cadherin (Angelucci et al., 2012; Blaschuk 2020). Tumors can be categorized based on the number of CAFs residing in their microenvironment (Bagaev et al., 2021). Solid tumors (e.g., pancreatic ductal adenocarcinoma) become fibrotic due to the large numbers of CAFs in their microenvironment secreting ECM components such as collagen (Öhlund et al., 2017; Winkler et al., 2020). CAFs are thus capable of regulating the immune response to a tumor by modulating direct interactions between T cells and cancer cells. Similarly, the ability of small molecules (e.g., anti-cancer drugs) and biologics (e.g., anti-tumor monoclonal antibodies) to penetrate the tumor and bind cancer cells is limited by permeability barriers arising from CAFs and their associated ECM components within the stroma (Olive et al., 2009; Joyce and Fearon 2015; Olive 2015). As N-cadherin antagonists (e.g., compound 15) block CAF adhesion and decrease their viability (Blaschuk 2020), they could be used to remodel fibrotic tumors, thus facilitating T cell-cancer cell interactions and drug delivery into tumors.

### N-Cadherin Antagonists as Modulators of Blood Vessel Formation, Stability, and Function

There are two types of mural cells comprising blood vessels: pericytes and VSMC (Smyth et al., 2018). The latter cell type is only present in large blood vessels (i.e., arteries, veins) (Smyth et al., 2018; Caporarello et al., 2019). The assembly and maintenance of a functional vasculature is dependent on N-cadherin-mediated EC-EC and EC-mural cell (e.g., pericyte) adhesion (Gerhardt et al., 2000; Devemy and Blaschuk 2008; Blaschuk and Devemy 2009; Caporarello et al., 2019; Kruse et al., 2019; Mrozik et al., 2020; Perrot et al., 2020). N-cadherin antagonists (e.g., ADH-1, LCRF-0006, anti-N-cadherin antibodies) can therefore be used to manipulate the vasculature, as they disrupt EC-EC and EC-pericyte interactions (Alexander et al., 1993; Blaschuk and Rowlands 2000; Gerhardt et al., 2000; Devemy and Blaschuk 2008; Caporarello et al., 2019; Mrozik et al., 2020).

Pericytes have emerged as key regulators of blood flow and vessel permeability (Gerhardt et al., 2000; Gerhardt and Betsholtz 2003; Blaschuk and Devemy 2009; Caruso et al., 2009; Caporarello et al., 2019; Kruse et al., 2019; Li et al., 2019; Perrot et al., 2020; Alarcon-Martinez et al., 2021). They stabilize blood vessels by adhering to ECs via AJs and secreting components (e.g., collagen) of the basement membrane. Furthermore, pericytes are contractile cells that ensheathe ECs, thus regulating blood flow (Caporarello et al., 2019; Perrot et al., 2020; Alarcon-Martinez et al., 2021). Blood vessels deficient in pericytes (e.g., tumor microvessels) exhibit poor blood flow, increased permeability, and an abnormal basement membrane (Jain 2005; Blaschuk and Devemy 2009; Li et al., 2019; Mrozik et al., 2020) making them particularly susceptible to disruption by N-cadherin antagonists (Blaschuk and Rowlands 2000, 2006). These antagonists can be considered as vasculature-targeting agents for use in the treatment of cancer.

The N-cadherin antagonist LCRF-0006 has been shown to disrupt EC adhesion and tube formation *in vitro* and increase tumor blood vessel permeability *in vivo* (Mrozik et al., 2020). This antagonist enhanced the potency of bortezomib (a drug used to treat multiple myeloma) in a mouse model of multiple myeloma. These observations suggest that N-cadherin antagonists can be used to promote drug penetration into tumors by lowering permeability barriers.

In addition to being modulators of blood vessel integrity (i.e., vasculature-targeting agents), N-cadherin antagonists can also inhibit blood vessel formation (i.e., vasculogenesis and angiogenesis) by preventing EC-EC and EC-pericyte adhesion (Radice et al., 1997; Blaschuk and Rowlands 2000, 2006; Gerhardt et al., 2000; Blaschuk et al., 2003; Paik et al., 2004). Studies have shown that tumors require an adequate blood supply to grow (Jain 2005; Li et al., 2018, Li et al., 2019). N-cadherin antagonists can conceivably act as anti-angiogenic agents to inhibit tumor growth. They might also be effective in treating a variety of angiogenic-dependent diseases (e.g., age-related macular degeneration, obesity) (Fallah et al., 2019).

A difficult concept to grasp is that N-cadherin antagonists can have both anti- and pro-angiogenic activities by virtue of their ability to regulate EC-pericyte adhesion (Gerhardt and Betsholtz 2003; Perrot et al., 2020; Payne et al., 2021). The process of angiogenesis has several distinct phases (Li et al., 2019; Payne et al., 2021). The first phase involves disengagement of the pericytes from the endothelium of mature microvessels. This process is facilitated by N-cadherin antagonists (Gerhardt et al., 2000; Gerhardt and Betsholtz 2003; Paik et al., 2004; Perrot et al., 2020) which enable the pericyte-free ECs to proliferate and migrate, thus beginning the formation of new microvessels at distant sites (Li et al., 2019; Payne et al., 2021). These antagonists could therefore be used to stimulate angiogenesis in damaged tissues. For example, ischemic stroke results in damage to the brain due to blood vessel occlusion (Yang et al., 2017; Freitas-Andrade et al., 2020; Di et al., 2021). Angiogenesis begins in the peri-infarct region rapidly after the stroke. N-cadherin antagonists could hasten the healing process by stimulating microvessel formation and neurogenesis. In addition, these antagonists could facilitate blood flow into the region by loosening adhesion between contracted pericytes and the endothelium thus allowing microvessels to become unconstricted (Freitas-Andrade et al., 2020; Di et al., 2021). Reducing pericyte contraction and thereby rapidly stimulating blood flow after ischemic stroke may lessen damage to the brain.

Uncoupling of microvascular pericyte-EC adhesion has also been shown to give rise to scar-forming stromal cells (i.e., fibroblasts) at sites of damage in the central nervous system (CNS) (Dias et al., 2021; Rentsch and Rust 2022). Specifically, a subpopulation of pericytes (termed type A pericytes) residing in the mammalian CNS vasculature disengages from the endothelium after either brain or spinal cord injury, proliferates, differentiates into scar-forming stromal cells (i.e., fibroblasts secreting collagen 1 and fibronectin), aggregates and migrates into the infarct. N-cadherin antagonists may prevent scar formation in the CNS lesions by inhibiting some, or all these processes. This hypothesis deserves examination.

VSMC proliferation and migration contributes to restenosis (i.e., reduction in blood vessel lumen diameter) after angioplasty, stent placement, bypass vein graft surgery and atherogenesis (Lyon et al., 2010; Wadey et al., 2018; Aoki and Tanabe 2021). N-cadherin and the β-catenin/Wnt signaling pathway have been shown to be directly involved in regulating VSMC proliferation and migration (Lyon et al., 2010; Wadey et al., 2018). The N-cadherin antagonist, ADH-1 is capable of inhibiting VSMC migration and promotes apoptosis of these cells (Lyon et al., 2010). N-cadherin antagonists may therefore be useful inhibitors of restenosis and could be incorporated into drug-eluting stents.

The multimodal actions of N-cadherin antagonists on blood vessels necessitates a careful evaluation of their biological activities on both normal vasculature, and that present in tumors and damaged tissues. Studies clearly indicate that these antagonists have different effects on normal, as opposed to tumor blood vessels. Normal vasculature does not appear to be severely affected by N-cadherin antagonists, whereas abnormal vasculature deficient in pericytes is sensitive to these agents (Beasley et al., 2009; Perotti et al., 2009; Tanaka et al., 2010; Mrozik et al., 2020). The effect of N-cadherin antagonists will likely be influenced by many variables including: the repertoire of CAMs (e.g., integrins, nectins) expressed by a particular cell type, method of administration, dosing schedule, tumor size and type, pericyte coverage of the endothelium, and growth factor (e.g., VEGF) levels. It is also important to consider the type of antagonist to be used for a particular application. Small molecule antagonists will have different pharmacokinetics than anti-N-cadherin antibodies (e.g., dissimilar half-lives in the blood). Judicious use of N-cadherin antagonists will be required to achieve optimal effects on the vasculature.

### N-Cadherin Antagonists as Cancer Therapeutics

The effects of N-cadherin antagonists on tumors are likely to be multifaceted and dependent on tumor type. N-cadherin antagonists can conceivably affect the tumor’s cancer cells, CAFs, pericytes and ECs, as they all express N-cadherin. The cellular compositions of solid (e.g., pancreatic ductal adenocarcinoma) and liquid (e.g., multiple myeloma) tumors are vastly different (Olive 2015; Mrozik et al., 2018; Li et al., 2019; Bagaev et al., 2021). N-cadherin antagonists would consequently be expected to have significantly different biological effects on solid and liquid tumors.

Aberrant N-cadherin expression has been observed in many cancer cell types (Nagi et al., 2005; Gravdal et al., 2007; Mrozik et al., 2018; Cao et al., 2019; Derynck and Weinberg 2019). N-cadherin is involved in regulating cancer cell adhesion, proliferation, survival, invasiveness, and metastasis. These observations suggest that N-cadherin antagonists could serve as potent anti-cancer drugs due to their ability to affect many of the steps necessary for tumor progression.

The biological effects of N-cadherin antagonists on tumors have been extensively studied in a variety of preclinical animal models (Shintani et al., 2008; Tanaka et al., 2010; Blaschuk 2015; Mrozik et al., 2018, 2020; Sun et al., 2021). These antagonists are capable of decreasing the growth of various cancer types in mice (e.g., pancreatic and prostate tumors). N-cadherin antagonists have also been shown to enhance the effectiveness of anti-cancer drugs (e.g., bortezomib) by increasing tumor blood vessel permeability (Mrozik et al., 2020). This observation suggests that N-cadherin antagonists would be useful in combination therapy to increase the penetration of drugs into tumors.

In addition, N-cadherin antagonists can potentiate the immune response to tumors by regulating programmed cell death ligand 1 (PD-L1) levels (Sun et al., 2021). PD-L1 is expressed on the surface of cancer cells, where it can interact with the receptor, PD-1 on the T cell membrane to suppress the immune response against tumors (Dong et al., 2018; Ribas and Wolchok 2018). ADH-1 is capable of down-regulating PD-L1 expression, thus facilitating T cell-mediated killing of cancer cells (Sun et al., 2021). The usefulness of N-cadherin antagonist in immuno-oncologic therapies has not been fully appreciated to date.

N-cadherin has recently emerged as a potential therapeutic target for three types of brain cancers: meningioma (Magill et al., 2020), neuroblastoma (Lammens et al., 2012) and glioblastoma multiforme (Smits et al., 2020). There are currently no adequate treatments for these cancers. N-cadherin antagonists have the potential to fulfill the need for new brain cancer therapeutics.

A particularly noteworthy study demonstrated that AJs containing the N-cadherin/β−catenin/p120 complex are necessary for the formation of migrating glioma cell collectives infiltrating the brain (Gritsenko et al., 2020). Down-regulation of either these proteins prevented glioblastoma cell adhesion and formation of cell collectives *in vitro*. Cell proliferation was also decreased by the down-regulation of p120 (N-cadherin and β-catenin were not examined). These observations are in concordance with those concerning the importance of N-cadherin in facilitating the collective migration of fibroblasts during the process of scar formation.

Studies have shown that the N-cadherin antagonists ADH-1 and compound 15 decrease glioblastoma and neuroblastoma cell viability *in vitro* (Smits et al., 2020; Lammens et al., 2012, respectively). ADH-1 has also been shown to inhibit the proliferation and tumorigenesis of meningioma cells in co-cultures with human cerebral organoids (Magill et al., 2020). Collectively, these observations indicate that N-cadherin antagonists may have clinical utility for the treatment of brain cancers.

As discussed previously, ADH-1 is the only N-cadherin antagonist that has entered clinical trials (Beasley et al., 2009; Perotti et al., 2009; Yarom et al., 2011, 2013). ADH-1 was well-tolerated, altered tumor vascular permeability and decreased tumor growth in some patients. For example, a female with an N-cadherin positive metastatic adrenocortical carcinoma was treated with a single dose of ADH-1 at 150 mg/mm2 (Yarom et al., 2011). This treatment caused transient normalization of plasma cortisol, tumor necrosis, and a reduction in tumor perfusion. In another Phase I clinical study, two patients with ovarian cancer exhibited prolonged disease stabilization and one patient with fallopian tube carcinoma had a mixed response to treatment with ADH-1 (Perotti et al., 2009). These results indicate that N-cadherin antagonists might be useful in the treatment of gynaecological cancers. The results obtained from clinical trials have encouraged the development of more potent, small molecule, N-cadherin antagonists (e.g., compound 15). These new antagonists have yet to be evaluated for their activity against tumors in preclinical and clinical studies.

### Biological Properties of N-Cadherin Agonists

Most N-cadherin agonists contain the N-cadherin CAR sequence, HAV (Utton et al., 2001; Williams et al., 2002; Skaper et al., 2004; Feng et al., 2020; Zhang et al., 2020; Dieterle et al., 2021). For example, the soluble cyclic peptide N-Ac-CHAVDINGHAVDIC-NH2 has been shown to be such an agonist (Williams et al., 2002; Skaper et al., 2004). This peptide can stimulate neurite outgrowth *in vitro*, a process known to be dependent on N-cadherin. The cyclic peptide is thought to cause dimerization of N-cadherin monomers within the plane of the plasma membrane, thus activating the associated FGF receptor signaling pathway.

Peptides containing the HAV sequence can be attached to scaffolds (e.g., methacrylated hyaluronic acid hydrogels) to form biomaterials that are able to act as N-cadherin agonists *in vitro* and *in vivo* (Bian et al., 2013; Feng et al., 2020; Zhang et al., 2020; Dieterle et al., 2021; Kaur and Roy 2021; Zhu et al., 2021). For example, human MSC displaying N-cadherin can be induced to enter chondrogenesis by culturing them in HAV-containing hydrogels (Li et al., 2017). The canonical Wnt/β−catenin signaling pathway is suppressed in these cultures causing a reduction of β-catenin levels in the nucleus (see preceding discussion of this pathway). In another example, a hydrogel composed of an HAV-containing peptide covalently tethered to polyethylene glycol dimethacrylate was shown to promote human neural stem cell differentiation, neurite outgrowth, and survival *in vitro* (Kaur and Roy 2021). These two examples demonstrate the ability of HAV-containing biomaterials to act as N-cadherin agonists and direct stem cell differentiation.

### Prospective Therapeutic Applications of N-Cadherin Agonists

The observation that soluble N-cadherin agonists and HAV-containing peptides tethered to hydrogels can promote certain steps necessary for neural regeneration suggests that they have the potential to serve as therapeutics for CNS and peripheral nervous system injuries and diseases. In particular, hydrogels with HAV-conjugated peptides have been shown to stimulate neurogenesis, axonal sprouting, and neural plasticity (Lim et al., 2017; Katoh et al., 2019; Kaur and Roy 2021). Biomaterials composed of HAV peptide-conjugated hydrogels have also shown promise in stimulating the regeneration of cartilage (Feng et al., 2020; Zhang et al., 2020). For example, bone marrow MSCs encapsulated in such hydrogels are capable of promoting cartilage formation in rabbit knee osteochondral defects (Feng et al., 2020). Implanting or injecting stem cells (e.g., MSCs) encapsulated in appropriately designed biomaterials (e.g., those conjugated with HAV-containing peptides) into damaged tissue represents a new approach to treating a variety of injuries.

### Summary and Future Directions

A number of N-cadherin agonists and antagonists have been discovered over the last 3 decades, but only one antagonist (the cyclic peptide CHAVC, designated ADH-1) has entered human clinical trials (Table 1). It was well tolerated and showed promise as an anti-cancer drug. More potent, orally available small molecules have recently been described (i.e., compound 15 and other derivatives of piperidin-4 amine). Their activities remain to be extensively evaluated in animal models for cancer and other maladies.

**TABLE 1:** N-cadherin antagonists and agonists.^a^

Compound name	Type	Structure	N-cadherin modulating activity	Stage of development	Therapeutic potential
HAV-containing peptide	linear peptide	LRAHAVDNG	antagonist	discovery	*in vitro* proof of concept
Trp-containing peptide	linear peptide	SWTLYTPSGQSK	antagonist	discovery	*in vitro* proof of concept
ADH-1 (Exherin)	cyclic peptide	CHAVC	antagonist	clinical (Phase 2)	cancer
LCRF-0006	small molecule	Triazole	antagonist	pre-clinical	drug delivery
Compound 15	small molecule	Piperidin-4-amine	antagonist	pre-clinical	pancreatic cancer
HAV dimeric	cyclic peptide	CHAVDINGHAVDIC	agonist	pre-clinical	neural regeneration
HAV-biomaterial	linear peptide conjugated to hydrogel	HAV peptide-hydrogel	agonist	pre-clinical	cartilage regeneration

a See text for details.

N-cadherin agonists (HAV-containing peptides conjugated to hydrogels) have demonstrated the ability to influence stem cell differentiation *in vitro* and *in vivo*. These biomaterials have shown promise for use in tissue regeneration and should be subjected to further studies.

A major theme that has emerged concerns the ability of N-cadherin antagonists to regulate fibrosis by virtue of their ability to inhibit fibroblast adhesion and collagen production. Preclinical studies have shown that ADH-1 can prevent fibroblast-dependent scar tissue formation in the skin. N-cadherin antagonists thus have the potential to serve as therapeutics for many fibrosis-dependent diseases.

N-cadherin agonists and antagonists will likely need to be combined with other agents (e.g., growth factors. T cells, anti-cancer drugs, monoclonal antibodies) to achieve maximum efficacy in the treatment of various maladies. Combination therapies involving N-cadherin function modulators remain to be extensively investigated.

## References

[B1] Adhikari S. Moran J. Weddle C. Hinczewski M. (2018). Unraveling the Mechanism of the Cadherin-Catenin-Actin Catch Bond. Plos Comput. Biol. 14, e1006399. 10.1371/journal.pcbi.1006399 30118477 PMC6114904

[B2] Alarcon-Martinez L. Yemisci M. Dalkara T. (2021). Pericyte Morphology and Function. Histol. Histopathol. 36, 633–643. 10.14670/HH-18-314 33595091

[B3] Alexander J. S. Blaschuk O. W. Haselton F. R. (1993). An N-cadherin-like Protein Contributes to Solute Barrier Maintenance in Cultured Endothelium. J. Cel. Physiol. 156, 610–618. 10.1002/jcp.1041560321 8360263

[B4] Aloysius A. DasGupta R. Dhawan J. (2018). The Transcription Factor Lef1 Switches Partners from β-catenin to Smad3 during Muscle Stem Cell Quiescence. Sci. Signal. 11, eaan3000. 10.1126/scisignal.aan3000 30042129

[B5] Anastasiadis P. Z. Reynolds A. B. (2001). Regulation of Rho GTPases by P120-Catenin. Curr. Opin. Cel Biol. 13, 604–610. 10.1016/s0955-0674(00)00258-1 11544030

[B6] Angelucci C. Maulucci G. Lama G. Proietti G. Colabianchi A. Papi M. (2012). Epithelial-stromal Interactions in Human Breast Cancer: Effects on Adhesion, Plasma Membrane Fluidity and Migration Speed and Directness. PLoS One 7, e50804. 10.1371/journal.pone.0050804 23251387 PMC3519494

[B7] Aoki J. Tanabe K. (2021). Mechanisms of Drug-Eluting Stent Restenosis. Cardiovasc. Interv. Ther. 36, 23–29. 10.1007/s12928-020-00734-7 33222019

[B8] Ascensión A. M. Fuertes-Álvarez S. Ibañez-Solé O. Izeta A. Araúzo-Bravo M. J. (2021). Human Dermal Fibroblast Subpopulations Are Conserved across Single-Cell RNA Sequencing Studies. J. Invest. Dermatol. 141, 1735–1744. e35. 10.1016/j.jid.2020.11.028 33385399

[B9] Bachir A. I. Horwitz A. R. Nelson W. J. Bianchini J. M. (2017). Actin-Based Adhesion Modules Mediate Cell Interactions with the Extracellular Matrix and Neighboring Cells. Cold Spring Harb. Perspect. Biol. 9, a023234. 10.1101/cshperspect.a023234 28679638 PMC5495059

[B10] Bagaev A. Kotlov N. Nomie K. Svekolkin V. Gafurov A. Isaeva O. (2021). Conserved Pan-Cancer Microenvironment Subtypes Predict Response to Immunotherapy. Cancer Cell 39, 845–865. e7. 10.1016/j.ccell.2021.04.014 34019806

[B11] Bays J. L. DeMali K. A. (2017). Vinculin in Cell-Cell and Cell-Matrix Adhesions. Cell. Mol. Life Sci. 74, 2999–3009. 10.1007/s00018-017-2511-3 28401269 PMC5501900

[B12] Beasley G. M. McMahon N. Sanders G. Augustine C. K. Selim M. A. Peterson B. (2009). A Phase 1 Study of Systemic ADH-1 in Combination with Melphalan via Isolated Limb Infusion in Patients with Locally Advanced In-Transit Malignant Melanoma. Cancer 115, 4766–4774. 10.1002/cncr.24509 19637344 PMC7533793

[B13] Bian L. Guvendiren M. Mauck R. L. Burdick J. A. (2013). Hydrogels that Mimic Developmentally Relevant Matrix and N-Cadherin Interactions Enhance MSC Chondrogenesis. Proc. Natl. Acad. Sci. 110, 10117–10122. 10.1073/pnas.1214100110 23733927 PMC3690835

[B14] Black M. Milewski D. Le T. Ren X. Xu Y. Kalinichenko V. V. (2018). FOXF1 Inhibits Pulmonary Fibrosis by Preventing CDH2-CDH11 Cadherin Switch in Myofibroblasts. Cel Rep. 23, 442–458. 10.1016/j.celrep.2018.03.067 PMC5947867 29642003

[B15] Blaschuk O. W. Devemy E. A-M. (2013). Compounds and Methods for Modulating Cadherin-Mediated Processes. U.S. Patent No 8,603,986 B2 (Washington, DC: U.S. Patent and Trademark Office).

[B16] Blaschuk O. W. Gour B. J. (2000). Compounds and Methods for Modulating Cell Adhesion. U.S. Patent No 6,031,072 (Washington, DC: U.S. Patent and Trademark Office).

[B17] Blaschuk O. W. Gour B. J. Farookhi R. Ali A. (2003). Compounds and Methods for Modulating Endothelial Cell Adhesion. U.S. Patent No 6,610,821 (Washington, DC: U.S. Patent and Trademark Office).

[B18] Blaschuk O. W. (2020). Modulators of Cell Adhesion, Methods and Compositions Therefor. U.S. Patent No 10,647,672 B2 (Washington, DC: U.S. Patent and Trademark Office).

[B19] Blaschuk O. W. Devemy E. (2009). Cadherins as Novel Targets for Anti-cancer Therapy. Eur. J. Pharmacol. 625, 195–1988. 10.1016/j.ejphar.2009.05.033 19836380

[B20] Blaschuk O. W. (2015). N-cadherin Antagonists as Oncology Therapeutics. Phil. Trans. R. Soc. B 370, 20140039. 10.1098/rstb.2014.0039 25533096 PMC4275908

[B21] Blaschuk O. W. Pouliot Y. Holland P. C. (1990a). Identification of a Conserved Region Common to Cadherins and Influenza Strain A Hemagglutinins. J. Mol. Biol. 211, 679–682. 10.1016/0022-2836(90)90065-t 2313692

[B22] Blaschuk O. W. Rowlands T. M. (2000). Cadherins as Modulators of Angiogenesis and the Structural Integrity of Blood Vessels. Cancer Metastasis Rev. 19, 1–5. 10.1023/a:1026522216059 11191048

[B23] Blaschuk O. W. Rowlands T. M. (2006). “Cadherin Antagonists as Vasculature-Targeting Agents,” in Vascular-targeted Therapies in Oncology. Editor Siemann D. W. (Hoboken NJ: John Wiley & Sons), 195–204. 10.1002/0470035439.ch11

[B24] Blaschuk O. W. Sullivan R. David S. Pouliot Y. (1990b). Identification of a Cadherin Cell Adhesion Recognition Sequence. Develop. Biol. 139, 227–229. 10.1016/0012-1606(90)90290-y 2328837

[B25] Brasch J. Harrison O. J. Ahlsen G. Carnally S. M. Henderson R. M. Honig B. (2011). Structure and Binding Mechanism of Vascular Endothelial Cadherin: a Divergent Classical Cadherin. J. Mol. Biol. 408, 57–73. 10.1016/j.jmb.2011.01.031 21269602 PMC3084036

[B26] Brasch J. Katsamba P. S. Harrison O. J. Ahlsén G. Troyanovsky R. B. Indra I. (2018). Homophilic and Heterophilic Interactions of Type II Cadherins Identify Specificity Groups Underlying Cell-Adhesive Behavior. Cel Rep. 23, 1840–1852. 10.1016/j.celrep.2018.04.012 PMC6029887 29742438

[B27] Bunse S. Garg S. Junek S. Vogel D. Ansari N. Stelzer E. H. K. (2013). Role of N-Cadherin Cis and Trans Interfaces in the Dynamics of Adherens Junctions in Living Cells. PLoS ONE 8, e81517. 10.1371/journal.pone.0081517 24312555 PMC3847041

[B28] Cadigan K. M. Waterman M. L. (20122012). TCF/LEFs and Wnt Signaling in the Nucleus. Cold Spring Harbor Perspect. Biol. 4, a007906. 10.1101/cshperspect.a007906 PMC3536346 23024173

[B29] Cao Z.-Q. Wang Z. Leng P. (2019). Aberrant N-Cadherin Expression in Cancer. Biomed. Pharmacother. 118, 109320. 10.1016/j.biopha.2019.109320 31545265

[B30] Caporarello N. D’Angeli F. Cambria M. T. Candido S. Giallongo C. Salmeri M. (2019). Pericytes in Microvessels: From "Mural" Function to Brain and Retina Regeneration. Ijms 20, 6351. 10.3390/ijms20246351 PMC6940987 31861092

[B32] Caruso R. A. Fedele F. Finocchiaro G. Pizzi G. Nunnari M. Gitto G. (2009). Ultrastructural Descriptions of Pericyte/endothelium Peg-Socket Interdigitations in the Microvasculature of Human Gastric Carcinomas. Anticancer Res. 29, 449–453. 19331185

[B33] Chanda D. Otoupalova E. Smith S. R. Volckaert T. De Langhe S. P. Thannickal V. J. (2019). Developmental Pathways in the Pathogenesis of Lung Fibrosis. Mol. Aspects Med. 65, 56–69. 10.1016/j.mam.2018.08.004 30130563 PMC6374163

[B34] Charras G. Yap A. S. (2018). Tensile Forces and Mechanotransduction at Cell-Cell Junctions. Curr. Biol. 28, R445–R457. 10.1016/j.cub.2018.02.003 29689229

[B35] Cheng S.-L. Lecanda F. Davidson M. K. Warlow P. M. Zhang S.-F. Zhang L. (1998). Human Osteoblasts Express a Repertoire of Cadherins, Which Are Critical for BMP-2-Induced Osteogenic Differentiation. J. Bone Miner. Res. 13, 633–644. 10.1359/jbmr.1998.13.4.633 9556063

[B36] Chuah M. I. David S. Blaschuk O. (1991). Differentiation and Survival of Rat Olfactory Epithelial Neurons in Dissociated Cell Culture. Develop. Brain Res. 60, 123–132. 10.1016/0165-3806(91)90040-p 1893561

[B37] Colás-Algora N. Millán J. (2019). How many Cadherins Do Human Endothelial Cells Express? Cel. Mol. Life Sci. 76, 1299–1317. 10.1007/s00018-018-2991-9 PMC11105309 30552441

[B38] Correa-Gallegos D. Jiang D. Christ S. Ramesh P. Ye H. Wannemacher J. (2019). Patch Repair of Deep Wounds by Mobilized Fascia. Nature 576, 287–292. 10.1038/s41586-019-1794-y 31776510

[B39] Cosgrove B. D. Mui K. L. Driscoll T. P. Caliari S. R. Mehta K. D. Assoian R. K. (2016). N-cadherin Adhesive Interactions Modulate Matrix Mechanosensing and Fate Commitment of Mesenchymal Stem Cells. Nat. Mater 15, 1297–1306. 10.1038/nmat4725 27525568 PMC5121068

[B40] Derynck R. Weinberg R. A. (2019). EMT and Cancer: More Than Meets the Eye. Develop. Cel 49, 313–316. 10.1016/j.devcel.2019.04.026 PMC7672963 31063750

[B41] Devemy E. Blaschuk O. W. (2009). Identification of a Novel Dual E- and N-Cadherin Antagonist. Peptides 30, 1539–1547. 10.1016/j.peptides.2009.05.010 19465078

[B42] Devemy E. Blaschuk O. W. (2008). Identification of a Novel N-Cadherin Antagonist. Peptides 29, 1853–1861. 10.1016/j.peptides.2008.06.025 18655820

[B43] Di Z. Wu X. Xie W. Lin X. (2021). Effect of Pericytes on Cerebral Microvasculature at Different Time Points of Stroke. Biomed. Res. Int. 2021, 1–10. 10.1155/2021/5281182 PMC8716223 34977241

[B44] Dias D. O. Kalkitsas J. Kelahmetoglu Y. Estrada C. P. Tatarishvili J. Holl D. (2021). Pericyte-derived Fibrotic Scarring Is Conserved across Diverse central Nervous System Lesions. Nat. Commun. 12, 5501. 10.1038/s41467-021-25585-5 34535655 PMC8448846

[B45] Dieterle M. P. Husari A. Rolauffs B. Steinberg T. Tomakidi P. (2021). Integrins, Cadherins and Channels in Cartilage Mechanotransduction: Perspectives for Future Regeneration Strategies. Expert Rev. Mol. Med. 23, 1–20. 10.1017/erm.2021.16 PMC8724267 34702419

[B46] Domke L. M. Rickelt S. Dörflinger Y. Kuhn C. Winter-Simanowski S. Zimbelmann R. (2014). The Cell-Cell Junctions of Mammalian Testes: I. The Adhering Junctions of the Seminiferous Epithelium Represent Special Differentiation Structures. Cell Tissue Res 357, 645–665. 10.1007/s00441-014-1906-9 24907851 PMC4148596

[B47] Dong P. Xiong Y. Yue J. Hanley S. J. B. Watari H. (2018). Tumor-Intrinsic PD-L1 Signaling in Cancer Initiation, Development and Treatment: Beyond Immune Evasion. Front. Oncol. 8, 386. 10.3389/fonc.2018.00386 30283733 PMC6156376

[B48] Elbadawy M. Usui T. Yamawaki H. Sasaki K. (2019). Emerging Roles of C-Myc in Cancer Stem Cell-Related Signaling and Resistance to Cancer Chemotherapy: A Potential Therapeutic Target against Colorectal Cancer. Ijms 20, 2340. 10.3390/ijms20092340 PMC6539579 31083525

[B49] Erez N. Bershadsky A. Geiger B. (2005). Signaling from Adherens-type Junctions. Eur. J. Cel Biol. 84, 235–244. 10.1016/j.ejcb.2004.12.007 15819404

[B50] Erez N. Zamir E. Gour B. J. Blaschuk O. W. Geiger B. (2004). Induction of Apoptosis in Cultured Endothelial Cells by a Cadherin Antagonist Peptide: Involvement of Fibroblast Growth Factor Receptor-Mediated Signalling. Exp. Cel Res. 294, 366–378. 10.1016/j.yexcr.2003.11.033 15023527

[B51] Fallah A. Sadeghinia A. Kahroba H. Samadi A. Heidari H. R. Bradaran B. (2019). Therapeutic Targeting of Angiogenesis Molecular Pathways in Angiogenesis-dependent Diseases. Biomed. Pharmacother. 110, 775–785. 10.1016/j.biopha.2018.12.022 30554116

[B52] Fardi M. Alivand M. Baradaran B. Farshdousti Hagh M. Solali S. (2019). The Crucial Role of ZEB2: From Development to Epithelial‐to‐mesenchymal Transition and Cancer Complexity. J. Cel Physiol 234, 14783–14799. 10.1002/jcp.28277 30773635

[B53] Feng X. Zhou T. Xu P. Ye J. Gou Z. Gao C. (2020). Enhanced Regeneration of Osteochondral Defects by Using an Aggrecanase-1 Responsively Degradable and N-Cadherin Mimetic Peptide-Conjugated Hydrogel Loaded with BMSCs. Biomater. Sci. 8, 2212–2226. 10.1039/d0bm00068j 32119015

[B54] Flinn M. A. Link B. A. O’Meara C. C. (2020). Upstream Regulation of the Hippo-Yap Pathway in Cardiomyocyte Regeneration. Semin. Cel Develop. Biol. 100, 11–19. 10.1016/j.semcdb.2019.09.004 PMC7263368 31606277

[B55] Franke W. W. Rickelt S. Barth M. Pieperhoff S. (2009). The Junctions that Don't Fit the Scheme: Special Symmetrical Cell-Cell Junctions of Their Own Kind. Cel Tissue Res 338, 1–17. 10.1007/s00441-009-0849-z PMC2760712 19680692

[B56] Freitas-Andrade M. Raman-Nair J. Lacoste B. (2020). Structural and Functional Remodeling of the Brain Vasculature Following Stroke. Front. Physiol. 11, 948. 10.3389/fphys.2020.00948 32848875 PMC7433746

[B57] Gerhardt H. Betsholtz C. (2003). Endothelial-pericyte Interactions in Angiogenesis. Cel Tissue Res. 314, 15–23. 10.1007/s00441-003-0745-x 12883993

[B58] Gerhardt H. Wolburg H. Redies C. (2000). N‐cadherin Mediates Pericytic‐endothelial Interaction during Brain Angiogenesis in the Chicken. Develop. Dyn. 218, 472–479. 10.1002/1097-0177(200007)218:3<472::AID-DVDY1008>3.0.CO;2-# 10878612

[B59] Goossens S. Vandamme N. Van Vlierberghe P. Berx G. (2017). EMT Transcription Factors in Cancer Development Re-evaluated: Beyond EMT and MET. Biochim. Biophys. Acta (Bba) - Rev. Cancer 1868, 584–591. 10.1016/j.bbcan.2017.06.006 28669750

[B60] Gour B. J. Blaschuk O. W. Ali A. Feng N. Chen Z. Michaud S. D. (2007). Peptidomimetic Modulators of Cell Adhesion. U.S. Patent No 7,268,115 (Washington DC: U.S. Patent and Trademark Office).

[B61] Gravdal K. Halvorsen O. J. Haukaas S. A. Akslen L. A. (2007). A Switch from E-Cadherin to N-Cadherin Expression Indicates Epithelial to Mesenchymal Transition and Is of strong and Independent Importance for the Progress of Prostate Cancer. Clin. Cancer Res. 13, 7003–7011. 10.1158/1078-0432.CCR-07-1263 18056176

[B62] Gritsenko P. G. Atlasy N. Dieteren C. E. J. Navis A. C. Venhuizen J.-H. Veelken C. (2020). p120-catenin-dependent Collective Brain Infiltration by Glioma Cell Networks. Nat. Cel Biol. 22, 97–107. 10.1038/s41556-019-0443-x PMC6952556 31907411

[B63] Gul I. S. Hulpiau P. Saeys Y. van Roy F. (2017). Evolution and Diversity of Cadherins and Catenins. Exp. Cel Res. 358, 3–9. 10.1016/j.yexcr.2017.03.001 28268172

[B64] Harrison O. J. Jin X. Hong S. Bahna F. Ahlsen G. Brasch J. (2011). The Extracellular Architecture of Adherens Junctions Revealed by crystal Structures of Type I Cadherins. Structure 19, 244–256. 10.1016/j.str.2010.11.016 21300292 PMC3070544

[B65] Hatta K. Takeichi M. (1986). Expression of N-Cadherin Adhesion Molecules Associated with Early Morphogenetic Events in Chick Development. Nature 320, 447–449. 10.1038/320447a0 3515198

[B66] Haÿ E. Lemonnier J. Modrowski D. Lomri A. Lasmoles F. Marie P. J. (2000). N- and E-Cadherin Mediate Early Human Calvaria Osteoblast Differentiation Promoted by Bone Morphogenetic Protein-2. J. Cel Physiol. 183, 117–128. 10.1002/(SICI)1097-4652(200004)183:1<117::AID-JCP14>3.0.CO;2-# 10699973

[B67] Hong J. Y. Oh I.-H. McCrea P. D. (2016). Phosphorylation and Isoform Use in P120-Catenin during Development and Tumorigenesis. Biochim. Biophys. Acta (Bba) - Mol. Cel Res. 1863, 102–114. 10.1016/j.bbamcr.2015.10.008 PMC4658284 26477567

[B68] Houschyar K. S. Tapking C. Borrelli M. R. Popp D. Duscher D. Maan Z. N. (2019). Wnt Pathway in Bone Repair and Regeneration - what Do We Know So Far. Front. Cel Dev. Biol. 6, 170. 10.3389/fcell.2018.00170 PMC6330281 30666305

[B69] Hua F. Shang S. Yang Y.-w. Zhang H.-z. Xu T.-l. Yu J.-j. (2019). TRIB3 Interacts with β-Catenin and TCF4 to Increase Stem Cell Features of Colorectal Cancer Stem Cells and Tumorigenesis. Gastroenterology 156, 708–721. e15. 10.1053/j.gastro.2018.10.031 30365932

[B70] Hua Y. Yang Y. Li Q. He X. Zhu W. Wang J. (2018). Oligomerization of Frizzled and LRP5/6 Protein Initiates Intracellular Signaling for the Canonical WNT/β-catenin Pathway. J. Biol. Chem. 293, 19710–19724. 10.1074/jbc.RA118.004434 30361437 PMC6314125

[B71] Huang X. Khoong Y. Han C. Su D. Ma H. Gu S. (2021). Targeting Dermal Fibroblast Subtypes in Antifibrotic Therapy: Surface Marker as a Cellular Identity or a Functional Entity? Front. Physiol. 12, 694605. 10.3389/fphys.2021.694605 34335301 PMC8319956

[B72] Hulpiau P. Gul I. S. van Roy F. (2013). New Insights into the Evolution of Metazoan Cadherins and Catenins. Prog. Mol. Biol. Trans. Sci. 116, 71–94. 10.1016/B978-0-12-394311-8.00004-2 23481191

[B73] Hulpiau P. van Roy F. (2009). Molecular Evolution of the Cadherin Superfamily. Int. J. Biochem. Cel Biol. 41, 349–369. 10.1016/j.biocel.2008.09.027 18848899

[B74] Huxham J. Tabariès S. Siegel P. M. (2021). Afadin (AF6) in Cancer Progression: A Multidomain Scaffold Protein with Complex and Contradictory Roles. Bioessays 43, 2000221. 10.1002/bies.202000221 33165933

[B75] Jain R. K. (2005). Normalization of Tumor Vasculature: an Emerging Concept in Antiangiogenic Therapy. Science 307, 58–62. 10.1126/science10.1126/science.1104819 15637262

[B77] Janson D. Saintigny G. Mahé C. Ghalbzouri A. E. (2013). Papillary Fibroblasts Differentiate into Reticular Fibroblasts after Prolongedin Vitroculture. Exp. Dermatol. 22, 48–53. 10.1111/exd.12069 23278894

[B78] Jiang D. Rinkevich Y. Correa-Gallegos D. Ramesh P. Kalgudde Gopal S. Wannemacher J. (2021). Converting Fibroblastic Fates Leads to Wound Healing without Scar. Sig Transduct Target. Ther. 6, 332. 10.1038/s41392-021-00738-6 PMC8410910 34471094

[B79] Jiang J. (2017). CK1 in Developmental Signaling. Curr. Top. Dev. Biol. 123, 303–329. 10.1016/bs.ctdb.2016.09.002 28236970 PMC5837819

[B80] Jiang L. Li J. Zhang C. Shang Y. Lin J. (2020). YAP-mediated C-rosstalk between the Wnt and Hippo S-ignaling P-athways (Review. Mol. Med. Rep. 22, 4101–4106. 10.3892/mmr.2020.11529 33000236

[B81] Joyce J. A. Fearon D. T. (2015). T Cell Exclusion, Immune Privilege, and the Tumor Microenvironment. Science 348, 74–80. 10.1126/science.aaa6204 25838376

[B82] Katoh H. Yokota K. Fehlings M. G. (2019). Regeneration of Spinal Cord Connectivity through Stem Cell Transplantation and Biomaterial Scaffolds. Front. Cel. Neurosci. 13, 248. 10.3389/fncel.2019.00248 PMC6563678 31244609

[B83] Katoh M. (2017). Canonical and Non-canonical WNT Signaling in Cancer Stem Cells and Their Niches: Cellular Heterogeneity, Omics Reprogramming, Targeted Therapy and Tumor Plasticity (Review). Int. J. Oncol. 51, 1357–1369. 10.3892/ijo.2017.4129 29048660 PMC5642388

[B84] Kaur H. Roy S. (2021). Designing Aromatic N-Cadherin Mimetic Short-Peptide-Based Bioactive Scaffolds for Controlling Cellular Behaviour. J. Mater. Chem. B 9, 5898–5913. 10.1039/d1tb00598g 34263278

[B85] Keil R. Schulz J. Hatzfeld M. (2013). p0071/PKP4, a Multifunctional Protein Coordinating Cell Adhesion with Cytoskeletal Organization. Biol. Chem. 394, 1005–1017. 10.1515/hsz-2013-0114 23640939

[B86] Knüppel L. Ishikawa Y. Aichler M. Heinzelmann K. Hatz R. Behr J. (2017). A Novel Antifibrotic Mechanism of Nintedanib and Pirfenidone. Inhibition of Collagen Fibril Assembly. Am. J. Respir. Cel Mol. Biol. 57, 77–90. 10.1165/rcmb.2016-0217OC 28257580

[B87] Kon E. Calvo-Jiménez E. Cossard A. Na Y. Cooper J. A. Jossin Y. (2019). N-cadherin-regulated FGFR Ubiquitination and Degradation Control Mammalian Neocortical Projection Neuron Migration. Elife 8, e47673. 10.7554/eLife.47673 31577229 PMC6786859

[B88] Konstantinova I. R. Pearce A. C. (2015). Treatment of Fibrosis. U.S. Patent No 20150050240 (Washington, DC: U.S. Patent and Trademark Office).

[B89] Kota P. Terrell E. M. Ritt D. A. Insinna C. Westlake C. J. Morrison D. K. (2019). M-Ras/Shoc2 Signaling Modulates E-Cadherin Turnover and Cell-Cell Adhesion during Collective Cell Migration. Proc. Natl. Acad. Sci. USA. 116, 3536–3545. 10.1073/pnas.1805919116 30808747 PMC6397545

[B90] Kourtidis A. Ngok S. P. Anastasiadis P. Z. (2013). p120 Catenin. Prog. Mol. Biol. Transl. Sci. 116, 409–432. 10.1016/B978-0-12-394311-8.00018-2 23481205 PMC4960658

[B91] Krishnamurthy N. Kurzrock R. (2018). Targeting the Wnt/beta-Catenin Pathway in Cancer: Update on Effectors and Inhibitors. Cancer Treat. Rev. 62, 50–60. 10.1016/j.ctrv.2017.11.002 29169144 PMC5745276

[B92] Kruse K. Lee Q. S. Sun Y. Klomp J. Yang X. Huang F. (2019). N-cadherin Signaling via Trio Assembles Adherens Junctions to Restrict Endothelial Permeability. J. Cel Biol. 218, 299–316. 10.1083/jcb.201802076 PMC6314553 30463880

[B93] Lammens T. Swerts K. Derycke L. De Craemer A. De Brouwer S. De Preter K. (2012). N-cadherin in Neuroblastoma Disease: Expression and Clinical Significance. PLoS One 7, e31206. 10.1371/journal.pone.0031206 22355346 PMC3280274

[B94] Li R. Xu J. Wong D. S. H. Li J. Zhao P. Bian L. (2017). Self-assembled N-Cadherin Mimetic Peptide Hydrogels Promote the Chondrogenesis of Mesenchymal Stem Cells through Inhibition of Canonical Wnt/β-Catenin Signaling. Biomaterials 145, 33–43. 10.1016/j.biomaterials.2017.08.031 28843065

[B95] Li S. Xu H.-X. Wu C.-T. Wang W.-Q. Jin W. Gao H.-L. (2019). Angiogenesis in Pancreatic Cancer: Current Research Status and Clinical Implications. Angiogenesis 22, 15–36. 10.1007/s10456-018-9645-2 30168025

[B96] Li T. Kang G. Wang T. Huang H. (2018). Tumor Angiogenesis and Anti-angiogenic G-ene T-herapy for C-ancer (Review). Oncol. Lett. 16, 687–702. 10.3892/ol.2018.8733 29963134 PMC6019900

[B97] Li Y. Merkel C. D. Zeng X. Heier J. A. Cantrell P. S. Sun M. (2019). The N-Cadherin Interactome in Primary Cardiomyocytes as Defined by Quantitative Proximity Proteomics. J. Cel Sci. 132, jcs221606. 10.1242/jcs.221606 PMC6382013 30630894

[B98] Lim H. J. Khan Z. Wilems T. S. Lu X. Perera T. H. Kurosu Y. E. (2017). Human Induced Pluripotent Stem Cell Derived Neural Stem Cell Survival and Neural Differentiation on Polyethylene Glycol Dimethacrylate Hydrogels Containing a Continuous Concentration Gradient of N-Cadherin Derived Peptide His-Ala-Val-Asp-Ile. ACS Biomater. Sci. Eng. 3, 776–781. 10.1021/acsbiomaterials.6b00745 33440502

[B99] Lyon C. A. Koutsouki E. Aguilera C. M. Blaschuk O. W. George S. J. (2010). Inhibition of N-Cadherin Retards Smooth Muscle Cell Migration and Intimal Thickening via Induction of Apoptosis. J. Vasc. Surg. 52, 1301–1309. 10.1016/j.jvs.2010.05.096 20630685 PMC2977853

[B100] MacDonald B. T. Tamai K. He X. (2009). Wnt/β-Catenin Signaling: Components, Mechanisms, and Diseases. Develop. Cel 17, 9–26. 10.1016/j.devcel.2009.06.016 PMC2861485 19619488

[B101] Macedo G. S. Alemar B. Ashton-Prolla P. (2019). Reviewing the Characteristics of BRCA and PALB2-Related Cancers in the Precision Medicine Era. Genet. Mol. Biol. 42 (1 Suppl. l), 215–231. 10.1590/1678-4685-GMB-2018-0104 31067289 PMC6687356

[B102] Magill S. T. Vasudevan H. N. Seo K. Villanueva-Meyer J. E. Choudhury A. John Liu S. (2020). Multiplatform Genomic Profiling and Magnetic Resonance Imaging Identify Mechanisms Underlying Intratumor Heterogeneity in Meningioma. Nat. Commun. 11, 4803. 10.1038/s41467-020-18582-7 32968068 PMC7511976

[B103] Makrigiannakis A. Coukos G. Christofidou-Solomidou M. Gour B. J. Radice G. L. Blaschuk O. (1999). N-Cadherin-Mediated Human Granulosa Cell Adhesion Prevents Apoptosis. Am. J. Pathol. 154, 1391–1406. 10.1016/S0002-9440(10)65393-X 10329592 PMC1866595

[B104] Marie P. J. (2002). Role of N-Cadherin in Bone Formation. J. Cel. Physiol. 190, 297–305. 10.1002/jcp.10073 11857445

[B105] McLachlan R. W. Yap A. S. (2007). Not so Simple: the Complexity of Phosphotyrosine Signaling at Cadherin Adhesive Contacts. J. Mol. Med. 85, 545–554. 10.1007/s00109-007-0198-x 17429596

[B106] Mege R. M. Goudou D. Diaz C. Nicolet M. Garcia L. Geraud G. (1992). N-cadherin and N-CAM in Myoblast Fusion: Compared Localisation and Effect of Blockade by Peptides and Antibodies. J. Cel Sci 103 (Pt 4), 897–906. 10.1242/jcs.103.4.897 1487503

[B107] Menke A. Giehl K. (2012). Regulation of Adherens Junctions by Rho GTPases and P120-Catenin. Arch. Biochem. Biophys. 524, 48–55. 10.1016/j.abb.2012.04.019 22583808

[B108] Mikels A. J. Nusse R. (2006). Wnts as Ligands: Processing, Secretion and Reception. Oncogene 25, 7461–7468. 10.1038/sj.onc.1210053 17143290

[B109] Miyatani S. Shimamura K. Hatta M. Nagafuchi A. Nose A. Matsunaga M. (1989). Neural Cadherin: Role in Selective Cell-Cell Adhesion. Science 245, 631–635. 10.1126/science.2762814 2762814

[B110] Mizutani K. Miyata M. Shiotani H. Kameyama T. Takai Y. (2021). Nectins and Nectin-like Molecules in Synapse Formation and Involvement in Neurological Diseases. Mol. Cell Neurosci. 115, 103653. 10.1016/j.mcn.2021.103653 34242750

[B111] Mora J. M. Fenwick M. A. Castle L. Baithun M. Ryder T. A. Mobberley M. (2012). Characterization and Significance of Adhesion and Junction-Related Proteins in Mouse Ovarian Follicles1. Biol. Reprod. 86 (153), 1–14. 10.1095/biolreprod.111.096156 22321830

[B112] Mrozik K. M. Blaschuk O. W. Cheong C. M. Zannettino A. C. W. Vandyke K. (2018). N-cadherin in Cancer Metastasis, its Emerging Role in Haematological Malignancies and Potential as a Therapeutic Target in Cancer. BMC Cancer 18, 939. 10.1186/s12885-018-4845-0 30285678 PMC6167798

[B113] Mrozik K. M. Cheong C. M. Hewett D. R. Noll J. E. Opperman K. S. Adwal A. (2020). LCRF‐0006, a Small Molecule Mimetic of the N‐cadherin Antagonist Peptide ADH‐1, Synergistically Increases Multiple Myeloma Response to Bortezomib. FASEB BioAdvances 2, 339–353. 10.1096/fba.2019-00073 32617520 PMC7325588

[B114] Mulholland D. J. Dedhar S. Coetzee G. A. Nelson C. C. (2005). Interaction of Nuclear Receptors with the Wnt/β-Catenin/Tcf Signaling Axis: Wnt You like to Know? Endocr. Rev 26, 898–915. 10.1210/er.2003-0034 16126938

[B115] Nagafuchi A. Takeichi M. (1988). Cell Binding Function of E-Cadherin Is Regulated by the Cytoplasmic Domain. EMBO J. 7 (12), 3679–3684. 10.1002/j.1460-2075.1988.tb03249.x 3061804 PMC454940

[B116] Nagi C. Guttman M. Jaffer S. Qiao R. Keren R. Triana A. (2005). N-cadherin Expression in Breast Cancer: Correlation with an Aggressive Histologic Variant - Invasive Micropapillary Carcinoma. Breast Cancer Res. Treat. 94, 225–235. 10.1007/s10549-005-7727-5 16258702

[B117] Nelson W. J. (2008). Regulation of Cell-Cell Adhesion by the Cadherin-Catenin Complex. Biochem. Soc. Trans. 36 (Pt 2), 149–155. 10.1042/BST0360149 18363555 PMC3368607

[B118] Nguyen T. Mège R. M. (2016). N-cadherin and Fibroblast Growth Factor Receptors Crosstalk in the Control of Developmental and Cancer Cell Migrations. Eur. J. Cel Biol. 95, 415–426. 10.1016/j.ejcb.2016.05.002 27320194

[B119] Niessen C. M. (2007). Tight Junctions/adherens Junctions: Basic Structure and Function. J. Invest. Dermatol. 127, 2525–2532. 10.1038/sj.jid.5700865 17934504

[B120] Nieto M. A. Huang R. Y.-J. Jackson R. A. Thiery J. P. (2016). Emt: 2016. Cell 166, 21–45. 10.1016/j.cell.2016.06.028 27368099

[B121] Nurmik M. Ullmann P. Rodriguez F. Haan S. Letellier E. (2020). In Search of Definitions: Cancer‐associated Fibroblasts and Their Markers. Int. J. Cancer 146, 895–905. 10.1002/ijc.32193 30734283 PMC6972582

[B122] Oberlender S. A. Tuan R. S. (1994). Expression and Functional Involvement of N-Cadherin in Embryonic Limb Chondrogenesis. Development 120, 177–187. 10.1242/dev.120.1.177 8119125

[B123] Öhlund D. Handly-Santana A. Biffi G. Elyada E. Almeida A. S. Ponz-Sarvise M. (2017). Distinct Populations of Inflammatory Fibroblasts and Myofibroblasts in Pancreatic Cancer. J. Exp. Med. 214, 579–596. 10.1084/jem.20162024 28232471 PMC5339682

[B124] Olive K. P. Jacobetz M. A. Davidson C. J. Gopinathan A. McIntyre D. Honess D. (2009). Inhibition of Hedgehog Signaling Enhances Delivery of Chemotherapy in a Mouse Model of Pancreatic Cancer. Science 324, 1457–1461. 10.1126/science.1171362 19460966 PMC2998180

[B125] Olive K. P. (2015). Stroma, Stroma Everywhere (Far More Than You Think). Clin. Cancer Res. 21, 3366–3368. 10.1158/1078-0432.CCR-15-0416 25979482 PMC7535083

[B126] Paik J.-H. Skoura A. Chae S.-S. Cowan A. E. Han D. K. Proia R. L. (2004). Sphingosine 1-phosphate Receptor Regulation of N-Cadherin Mediates Vascular Stabilization. Genes Dev. 18, 2392–2403. 10.1101/gad.1227804 15371328 PMC522989

[B127] Patel S. D. Ciatto C. Chen C. P. Bahna F. Rajebhosale M. Arkus N. (2006). Type II Cadherin Ectodomain Structures: Implications for Classical Cadherin Specificity. Cell 124, 1255–1268. 10.1016/j.cell.2005.12.046 16564015

[B128] Payne L. B. Darden J. Suarez-Martinez A. D. Zhao H. Hendricks A. Hartland C. (2021). Pericyte Migration and Proliferation Are Tightly Synchronized to Endothelial Cell Sprouting Dynamics. Integr. Biol. (Camb) 13, 31–43. 10.1093/intbio/zyaa027 33515222 PMC7919101

[B129] Perotti A. Sessa C. Mancuso A. Noberasco C. Cresta S. Locatelli A. (2009). Clinical and Pharmacological Phase I Evaluation of Exherin (ADH-1), a Selective Anti-N-cadherin Peptide in Patients with N-Cadherin-Expressing Solid Tumours. Ann. Oncol. 20, 741–745. 10.1093/annonc/mdn695 19190075

[B130] Perrot C. Y. Herrera J. L. Fournier-Goss A. E. Komatsu M. (2020). Prostaglandin E2 Breaks Down Pericyte-Endothelial Cell Interaction via EP1 and EP4-dependent Downregulation of Pericyte N-Cadherin, Connexin-43, and R-Ras. Sci. Rep. 10, 1011186. 10.1038/s41598-020-68019-w PMC7341885 32636414

[B131] Pierre C. C. Hercules S. M. Yates C. Daniel J. M. (2019). Dancing from Bottoms up - Roles of the POZ-ZF Transcription Factor Kaiso in Cancer. Biochim. Biophys. Acta (Bba) - Rev. Cancer 1871, 64–74. 10.1016/j.bbcan.2018.10.005 PMC6467064 30419310

[B132] Plikus M. V. Wang X. Sinha S. Forte E. Thompson S. M. Herzog E. L. (2021). Fibroblasts: Origins, Definitions, and Functions in Health and Disease. Cell 184, 3852–3872. 10.1016/j.cell.2021.06.024 34297930 PMC8566693

[B133] Pobbati A. V. Hong W. (2020). A Combat with the YAP/TAZ-TEAD Oncoproteins for Cancer Therapy. Theranostics 10, 3622–3635. 10.7150/thno.40889 32206112 PMC7069086

[B134] Quintanal-Villalonga Á. Ferrer I. Guruceaga E. Cirauqui C. Marrugal Á. Ojeda L. (2020). FGFR1 and FGFR4 Oncogenicity Depends on N-Cadherin and Their Co-expression May Predict FGFR-Targeted Therapy Efficacy. EbioMedicine 53, 102683. 10.1016/j.ebiom.2020.102683 32114392 PMC7047190

[B135] Radice G. L. Rayburn H. Matsunami H. Knudsen K. A. Takeichi M. Hynes R. O. (1997). Developmental Defects in Mouse Embryos Lacking N-Cadherin. Develop. Biol. 181, 64–78. 10.1006/dbio.1996.8443 9015265

[B136] Ramakrishnan A.-B. Sinha A. Fan V. B. Cadigan K. M. (2018). The Wnt Transcriptional Switch: TLE Removal or Inactivation? BioEssays 40, 1700162. 10.1002/bies.201700162 PMC7495026 29250807

[B137] Rentsch N. H. Rust R. (2022). 'Scary' Pericytes: the Fibrotic Scar in Brain and Spinal Cord Lesions. Trends Neurosciences 45, 6–7. 10.1016/j.tins.2021.10.013 34774344

[B138] Ribas A. Wolchok J. D. (2018). Cancer Immunotherapy Using Checkpoint Blockade. Science 359, 1350–1355. 10.1126/science.aar4060 29567705 PMC7391259

[B139] Sahai E. Astsaturov I. Cukierman E. DeNardo D. G. Egeblad M. Evans R. M. (2020). A Framework for Advancing Our Understanding of Cancer-Associated Fibroblasts. Nat. Rev. Cancer 20, 174–186. 10.1038/s41568-019-0238-1 31980749 PMC7046529

[B140] Santiago L. Daniels G. Wang D. Deng F. M. Lee P. (2017). Wnt Signaling Pathway Protein LEF1 in Cancer, as a Biomarker for Prognosis and a Target for Treatment. Am. J. Cancer Res. 7, 1389–1406. 28670499 PMC5489786

[B141] Schnädelbach O. Blaschuk O. W. Symonds M. Gour B. J. Doherty P. Fawcett J. W. (2000). N-cadherin Influences Migration of Oligodendrocytes on Astrocyte Monolayers. Mol. Cell Neurosci. 15, 288–302. 10.1006/mcne.1999.0819 10736205

[B142] Shao X. Kang H. Loveless T. Lee G. R. Seok C. Weis W. I. (2017). Cell-cell Adhesion in Metazoans Relies on Evolutionarily Conserved Features of the α-catenin·β-catenin-binding Interface. J. Biol. Chem. 292, 16477–16490. 10.1074/jbc.M117.795567 28842483 PMC5633108

[B143] Shapiro L. Weis W. I. (2009). Structure and Biochemistry of Cadherins and Catenins. Cold Spring Harbor Perspect. Biol. 1, a003053. 10.1101/cshperspect.a003053 PMC2773639 20066110

[B144] Sharma M. Castro-Piedras I. Simmons G. E. Jr Pruitt K. (2018). Dishevelled: A Masterful Conductor of Complex Wnt Signals. Cell Signal. 47, 52–64. 10.1016/j.cellsig.2018.03.004 29559363 PMC6317740

[B146] Shimono Y. Rikitake Y. Mandai K. Mori M. Takai Y. (2012). Immunoglobulin Superfamily Receptors and Adherens Junctions. Subcell. Biochem. 60, 137–170. 10.1007/978-94-007-4186-7_7 22674071

[B147] Shintani Y. Fukumoto Y. Chaika N. Grandgenett P. M. Hollingsworth M. A. Wheelock M. J. (2008). ADH-1 Suppresses N-cadherin-dependent Pancreatic Cancer Progression. Int. J. Cancer 122, 71–77. 10.1002/ijc.23027 17721921

[B148] Skaper S. D. Facci L. Williams G. Williams E.-J. Walsh F. S. Doherty P. (2004). A Dimeric Version of the Short N-Cadherin Binding Motif HAVDI Promotes Neuronal Cell Survival by Activating an N-Cadherin/fibroblast Growth Factor Receptor Signalling cascade. Mol. Cell Neurosci. 26, 17–23. 10.1016/j.mcn.2003.12.015 15121175

[B149] Smits I. P. M. Blaschuk O. W. Willerth S. M. (2020). Novel N-Cadherin Antagonist Causes Glioblastoma Cell Death in a 3D Bioprinted Co-culture Model. Biochem. Biophysical Res. Commun. 529, 162–168. 10.1016/j.bbrc.2020.06.001 32703405

[B150] Smyth L. C. D. Rustenhoven J. Scotter E. L. Schweder P. Faull R. L. M. Park T. I. H. (2018). Markers for Human Brain Pericytes and Smooth Muscle Cells. J. Chem. Neuroanat. 92, 48–60. 10.1016/j.jchemneu.2018.06.001 29885791

[B151] Sun Y. Jing J. Xu H. Xu L. Hu H. Tang C. (2021). N-cadherin Inhibitor Creates a Microenvironment that Protect TILs from Immune Checkpoints and Treg Cells. J. Immunother. Cancer 9, e002138. 10.1136/jitc-2020-002138 33692219 PMC7949480

[B152] Szulzewsky F. Holland E. C. Vasioukhin V. (2021). YAP1 and its Fusion Proteins in Cancer Initiation, Progression and Therapeutic Resistance. Develop. Biol. 475, 205–221. 10.1016/j.ydbio.2020.12.018 33428889 PMC8107117

[B153] Takai Y. Nakanishi H. (2003). Nectin and Afadin: Novel Organizers of Intercellular Junctions. Cell Sci 1116 (Pt 1), 17–27. 10.1242/jcs.00167 12456712

[B154] Tanaka H. Kono E. Tran C. P. Miyazaki H. Yamashiro J. Shimomura T. (2010). Monoclonal Antibody Targeting of N-Cadherin Inhibits Prostate Cancer Growth, Metastasis and Castration Resistance. Nat. Med. 16, 1414–1420. 10.1038/nm.2236 21057494 PMC3088104

[B155] Taulet N. Comunale F. Favard C. Charrasse S. Bodin S. Gauthier-Rouvière C. (2009). N-cadherin/p120 Catenin Association at Cell-Cell Contacts Occurs in Cholesterol-Rich Membrane Domains and Is Required for RhoA Activation and Myogenesis. J. Biol. Chem. 284 (34), 23137–23145. 10.1074/jbc.M109.017665 19546217 PMC2755719

[B156] Ting L. H. Jahn J. R. Jung J. I. Shuman B. R. Feghhi S. Han S. J. (2012). Flow Mechanotransduction Regulates Traction Forces, Intercellular Forces, and Adherens Junctions. Am. J. Physiology-Heart Circulatory Physiol. 302, H2220–H2229. 10.1152/ajpheart.00975.2011 PMC3378295 22447948

[B157] Tsuchiya B. Sato Y. Kameya T. Okayasu I. Mukai K. (2006). Differential Expression of N-Cadherin and E-Cadherin in normal Human Tissues. Arch. Histology Cytol. 69, 135–145. 10.1679/aohc.69.135 16819153

[B158] Tuan R. S. (2003). Cellular Signaling in Developmental Chondrogenesis. The J. Bone Jt. Surgery-American Volume 85 (Suppl. 2), 137–141. 10.2106/00004623-200300002-00019 12721357

[B159] Utton M. A. Eickholt B. Howell F. V. Wallis J. Doherty P. (2001). Soluble N-Cadherin Stimulates Fibroblast Growth Factor Receptor Dependent Neurite Outgrowth and N-Cadherin and the Fibroblast Growth Factor Receptor Co-cluster in Cells. J. Neurochem. 76, 1421–1430. 10.1046/j.1471-4159.2001.00140.x 11238727

[B160] Vaisburg A. Blaschuk O. W. (2019). Modulators of Cell Adhesion, Methods and Compositions Therefor. U.S. Patent No 10,494,345 (Washington, DC: U.S. Patent and Trademark Offfice).

[B161] Valenta T. Hausmann G. Basler K. (2012). The many Faces and Functions of β-catenin. EMBO J. 31, 2714–2736. 10.1038/emboj.2012.150 22617422 PMC3380220

[B162] Vendome J. Felsovalyi K. Song H. Yang Z. Jin X. Brasch J. (2014). Structural and Energetic Determinants of Adhesive Binding Specificity in Type I Cadherins. Proc. Natl. Acad. Sci. USA 111 (111), E4175–E4184. 10.1073/pnas.1416737111 25253890 PMC4210030

[B163] Vite A. Radice G. L. (2014). N-cadherin/catenin Complex as a Master Regulator of Intercalated Disc Function. Cel Commun. Adhes. 21, 169–179. 10.3109/15419061.2014.908853 PMC6054126 24766605

[B164] Volk T. Cohen O. Geiger B. (1987). Formation of Heterotypic Adherens-type Junctions between L-CAM-Containing Liver Cells and A-CAM-Containing Lens Cells. Cell 50, 987–994. 10.1016/0092-8674(87)90525-3 3621349

[B165] Volk T. Geiger B. (1986b). A-CAM: a 135-kD Receptor of Intercellular Adherens Junctions. II. Antibody-Mediated Modulation of junction Formation. J. Cel Biol. 103, 1451–1464. 10.1083/jcb.103.4.1451 PMC2114343 3095334

[B166] Volk T. Geiger B. (1986a). A-CAM: a 135-kD Receptor of Intercellular Adherens Junctions. I. Immunoelectron Microscopic Localization and Biochemical Studies. J. Cel. Biol. 103, 1441–1450. 10.1083/jcb.103.4.1441 PMC2114326 3533954

[B167] Wadey K. Lopes J. Bendeck M. George S. (2018). Role of Smooth Muscle Cells in Coronary Artery Bypass Grafting Failure. Cardiovasc. Res. 114, 601–610. 10.1093/cvr/cvy021 29373656 PMC5852543

[B168] Wilby M. J. Muir E. M. Fok-Seang J. Gour B. J. Blaschuk O. W. Fawcett J. W. (1999). N-cadherin Inhibits Schwann Cell Migration on Astrocytes. Mol. Cell Neurosci. 14, 66–84. 10.1006/mcne.1999.0766 10433818

[B169] Williams E. Williams G. Gour B. J. Blaschuk O. W. Doherty P. (2000). A Novel Family of Cyclic Peptide Antagonists Suggests that N-Cadherin Specificity Is Determined by Amino Acids that Flank the HAV Motif. J. Biol. Chem. 275, 4007–4012. 10.1074/jbc.275.6.4007 10660557

[B170] Williams G. Williams E.-J. Doherty P. (2002). Dimeric Versions of Two Short N-Cadherin Binding Motifs (HAVDI and INPISG) Function as N-Cadherin Agonists. J. Biol. Chem. 277, 4361–4367. 10.1074/jbc.M109185200 11726665

[B171] Winkler J. Abisoye-Ogunniyan A. Metcalf K. J. Werb Z. (2020). Concepts of Extracellular Matrix Remodelling in Tumour Progression and Metastasis. Nat. Commun. 11, 5120. 10.1038/s41467-020-18794-x 33037194 PMC7547708

[B172] Yang S. Jin H. Zhu Y. Wan Y. Opoku E. N. Zhu L. (2017). Diverse Functions and Mechanisms of Pericytes in Ischemic Stroke. Cn 15, 892–905. 10.2174/1570159X15666170112170226 PMC5652032 28088914

[B173] Yang X. Lee S. H. (2016). Identification of ESE1 as a β-Catenin Binding Protein. Anticancer Res. 36, 2697–2703. 27272778

[B174] Yarom N. Stewart D. Avruch L. Malik R. Wells J. Jonker D. J. (2011). ADH-1 in the Treatment of Metastatic Adrenocortical Carcinoma-Ccase Report. Anticancer Res. 31, 3921–3925. 22110220

[B175] Yarom N. Stewart D. Malik R. Wells J. Avruch L. J. Jonker D. (2013). Phase I Clinical Trial of Exherin (ADH-1) in Patients with Advanced Solid Tumors. Ccp 8, 81–88. 10.2174/1574884711308010011 22280327

[B176] Young K. A. Biggins L. Sharpe H. J. (2021). Protein Tyrosine Phosphatases in Cell Adhesion. Biochem. J. 478, 1061–1083. 10.1042/BCJ20200511 33710332 PMC7959691

[B177] Zhang Y. Qin Z. Qu Z. Z. Ge M. Yang J. (2020). Cadherin-based Biomaterials: Inducing Stem Cell Fate towards Tissue Construction and Therapeutics. Prog. Nat. Sci. Mater. Int. 30, 597–608. 10.1016/j.pnsc.2020.09.001

[B178] Zhu M. Zhong W. Cao W. Zhang Q. Wu G. (2022). Chondroinductive/chondroconductive Peptides and Their-Functionalized Biomaterials for Cartilage Tissue Engineering. Bioactive Mater. 9, 221–238. 10.1016/j.bioactmat.2021.07.004 PMC8585793 34820567

